# Supplementary figures and images for: Overexpression of the White Opaque Switching Master Regulator Wor1 Alters Lipid Metabolism and Mitochondrial Function in Candida albicans
Source: J Fungi (Basel). 2022 Sep 28;8(10):1028. doi: 10.3390/jof8101028 (PMC9604646; doi:10.3390/jof8101028)

**A**

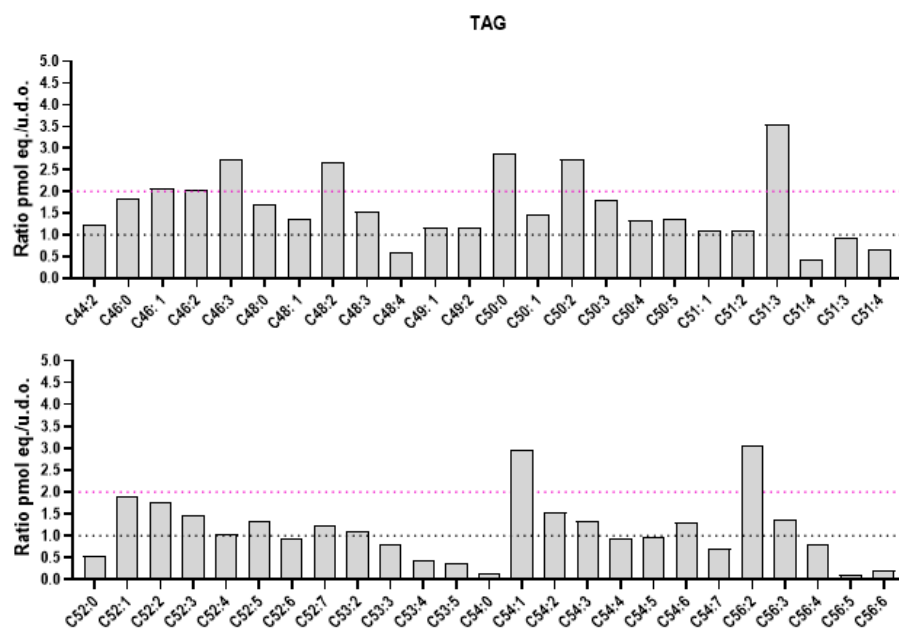

**B**

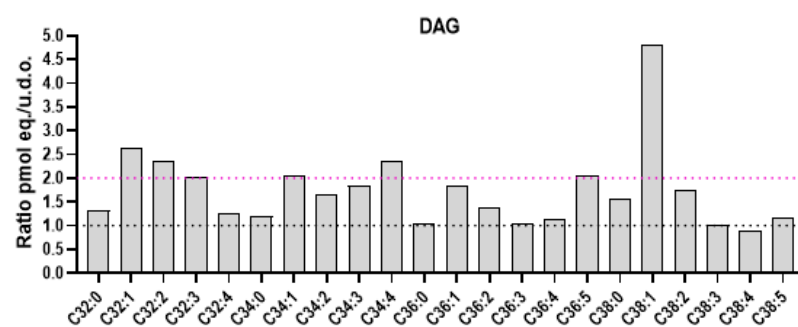

**Figure S1.** Total triacylglycerol (TAG) and diacylglycerol (DAG) content.

Supplement: Supplementary file 1 [file jof-08-01028-s001.zip › jof-1863998-supplementary.pdf]
